# A study on the refined population spatialization method integrating multi-source data: A case study of Yuxi city

**DOI:** 10.1371/journal.pone.0340430

**Published:** 2026-01-23

**Authors:** Fanghao Zhang, Feirui Jiang, Yuanshuo Zhang, Junzu Xu, Yang Ye, Zhaoliang Jia

**Affiliations:** Yunnan Earthquake Agency, Kunming, China; Ain Shams University, EGYPT

## Abstract

The precise assessment of earthquake disasters requires high accuracy in population spatialization data. With the rapid development of technologies such as remote sensing and social sensing (social perception), and the growing availability of large-scale geographic data, high-resolution remote sensing data and vast geographical spatial data provide new opportunities for inferring more accurate population data. This paper proposes a method that integrates social perception big data, building attribute data, and multi-source remote sensing data. Using Random Forest, XGBoost, LGBM, Gradient Boosting, AdaBoost, and CatBoost models as base learners, and RidgeCV regression as the secondary learner, a higher-performing Stacking ensemble learning prediction model is constructed. The method system is employed to spatialize the population data of Yuxi City for the year 2020 at a 50m resolution using the Partition Density Mapping technique. The results show that: (1) The population estimation model proposed in this paper outperforms the WorldPop dataset and LandScan dataset by more than 40% in terms of accuracy at the administrative village scale, providing higher simulation accuracy for regions with different population densities. This result highlights the refined spatial features of the population depicted in satellite remote sensing images, offering richer and more realistic population distribution information. (2) By measuring the SHAP feature contributions and importance of the variables, it is found that residential building area, POI, and nighttime light intensity are the most significant indicators of population distribution in Yuxi City, suggesting a higher correlation of social factors in population spatial redistribution.

## 1. Introduction

In earthquake disaster assessments, early evaluations often used population data aggregated at administrative units, such as through census or sampling surveys, which disconnected from the spatial features of the regions, failing to accurately reflect the population’s spatial distribution [1]. The main solution to these problems is the spatialization of population data, which involves using statistical data and qualitative or quantitative models to infer the distribution of population over time and space. This process enables the conversion and estimation of spatial units at different scales. With the rapid development of technologies like Earth observation, modern information networks, and geographic big data analysis (including remote sensing and social perception), the acquisition of large-scale disaster exposure and vulnerability parameters has become quicker and more accurate, providing rich and diverse data resources. “Economic and social data spatialization” has gradually become a research hotspot in academia [2], with population data spatialization being one of the critical research directions, where distributing statistical populations into finer spatial units is crucial for precise earthquake disaster risk assessments.

In recent years, various methods have been developed to decompose census data into grid units, such as area-weighted [3], geographically weighted regression [4], and partition density mapping [5], resulting in several large-scale gridded population datasets, including Gridded Population of the World [6], LandScan [7], WorldPop [8], and the Chinese Population Spatial Distribution Kilometer Grid Data [9]. However, most of these datasets have a spatial resolution of 1 km, which does not meet the needs of refined-scale studies, particularly in the precise evaluation of earthquake disaster risks. The WorldPop dataset, although the highest resolution open-source population dataset available, offers 100m resolution population grid data for regions in Central and South America, Africa, and Asia. However, due to the lower quality of open-source crowd-sourced OpenStreetMap data in many developing countries like China, its accuracy still has substantial room for improvement [10].

Population distribution is influenced by a variety of factors such as the environment, transportation, and resources. Combining relevant auxiliary data helps achieve accurate grid-based population distribution [8]. Remote sensing satellite imagery is widely used in population spatialization studies due to its high spatial resolution and short collection cycles. Currently, population spatialization modeling data has gradually become more diversified, refined, and dynamic. Some studies have integrated multiple data sources, such as land use and nighttime light data, to improve the accuracy of population estimation. However, these methods still face some challenges, such as land use interpretation data, which describes the spatial extent of population distribution but does not effectively capture population density variations within the same land type [11]. Nighttime light remote sensing data can to some extent differentiate the heterogeneity of population distribution, reflecting population density [12]. However, the light overflow effect caused by unrelated light sources, such as streetlights and construction sites, can lead to population misallocation [13]. Therefore, remote sensing and GIS-based population spatialization methods typically rely on multi-source data fusion. However, the main auxiliary data sources used, such as land use and nighttime light data, often have low spatial resolution, resulting in homogeneity problems at smaller scale units, limiting their ability to depict detailed differences in population distribution. This necessitates the integration of finer-grained auxiliary data for more precise population spatialization. Fine-grained social perception data, especially Points of Interest (POI), which contain precise locations and rich spatial semantics, are becoming a core data source for refined population modeling due to their widespread availability and their ability to reflect underlying human activities [14].

Notably, there exists a complex nonlinear relationship between population distribution and various influencing factors. Therefore, simulating refined population spatial distribution remains a significant challenge. In recent years, machine learning algorithms such as Random Forest [8,15–17], neural networks [18], and XG-Boost [19] have been applied in population spatialization simulations, showing significant improvements in simulation accuracy compared to traditional linear regression models [20]. However, different machine learning models exhibit considerable differences in terms of sensitivity to outliers, extrapolation ability, generalization, and overfitting [21]. Ensemble learning algorithms can combine multiple learners with varying strengths and weaknesses through various strategies, achieving significantly superior generalization performance compared to single models, making them better suited for complex problem modeling.

This paper uses administrative census data, building attribute data from the first national comprehensive disaster risk survey, POI data from Gaode Map, nighttime light intensity data, road data, and DEM and slope data as fine-grained geographic features to explore a refined population spatialization method for larger-scale regions based on an ensemble model of various machine learning algorithms. The study focuses on Yuxi City, where a population spatialization dataset at a 50m grid resolution is generated, and the method’s effectiveness is tested at the community administrative unit level. Through this research, it is expected to provide more accurate data support for grid-based population studies in earthquake emergencies, further improving the precision of rapid earthquake disaster assessments and providing more scientific and effective decision-making for earthquake emergency responses.

## 2. Overview of the study area and data sources

### 2.1. Study area

Yuxi City is a prefecture-level city in Yunnan Province, People’s Republic of China, located in the central part of Yunnan. Yuxi City has a total area of 15,300 square kilometers, consisting of 2 districts and 7 counties. The terrain of Yuxi is characterized by a high northwest and low southeast, with a tilt from northwest to southeast. The mountain ranges align with the structural lines, resulting in a complex landscape with mountains, valleys, plateaus, and basins interwoven. The region is located on a low-latitude plateau, and the alternating influence of two distinct atmospheric circulations during the winter and summer seasons leads to a significant monsoon climate. The region’s complex topography and significant elevation differences contribute to a wide variety of climate types, ranging from tropical to temperate zones. The region’s geographical location, geological conditions, ecological environment, and cultural factors collectively determine the characteristics of natural disasters, which include earthquakes, droughts, floods, landslides, hailstorms, and frost. The unique topography causes population concentration in the fault lake basin and Yuxi Basin. After disasters, these areas experience significant human and economic losses. Therefore, studying the population distribution characteristics in Yuxi can provide technical support for disaster risk assessment and relief in the region.

### 2.2. Data collection and processing

This study uses twelve datasets, including demographic data, administrative boundaries, building patch data, POI, Digital Elevation Model (DEM), slope, nighttime lights, road data, Satellite imagery data, WorldPop dataset, GPWv4 dataset, and Landscan-hd dataset. Detailed information is provided in Table 1 below.

**Table 1:** Datasets Used in This Study.

Data Category	Dataset Name	Year	Type	Resolution	Source	Description
Demographic	Population Data	2020	Table	–	Seventh National Population Census of Yuxi City	Population of each administrative village in Yuxi
Map division	Administrative Boundaries	2020	Vector Polygon	–	Yunnan Province Earthquake Disaster Defense Service Platform	Administrative boundaries of districts and counties in Yuxi City
Village Boundaries	Administrative Village Boundaries	2020	Vector Polygon	–	Yunnan Province Earthquake Disaster Defense Service Platform	Vector boundaries of administrative villages in Yuxi
Building Data	Building Patch Data	2020	Vector Polygon	–	The preliminary assessment of the seismic fortification capacity of buildings in Yuxi City	Building function, area, height, number of floors, age
POI	Points of Interest (POI) Data	2020	Vector Points	–	Gaode Map Development Platform	18 categories of POI data, including services like food, shopping, healthcare, etc.
Elevation	Elevation Data	2020	Raster	30m	NASA Earthdata DEM Products	Digital elevation model for Yuxi
Slope	Slope Data	2020	Raster	30m	Extracted from DEM Data	Slope data derived from the DEM
Nighttime Light	Nighttime Light Data	2020	Raster	500m	“NPP-VIIRS” Nighttime Light Dataset [22]	Nighttime lights data for Yuxi
Road Data	Road Data	2020	Vector Line	–	OSM OpenStreetMap	Road data from OSM platform for Yuxi
Comparison Data	Satellite imagery data	2020	image	–	Landsat-8 OLI data were obtained from the United States Geological Survey (USGS)	
Comparison Data	WorldPop	2020	Raster	100m	WorldPop Project	Comparison dataset for validating method effectiveness
Comparison Data	GPWv4	2020	Raster	30 arc seconds (approximately 1000m at the equator)	NASA Socioeconomic Data and Applications Center	Comparison dataset for validating method effectiveness
Comparison Data	Landscan-hd	2020	Raster	3 arc seconds (approximately 90m)	Oak Ridge National Laboratory Official Website	Comparison dataset for validating method effectiveness

## 3. Research methodology

**Fig 1:**
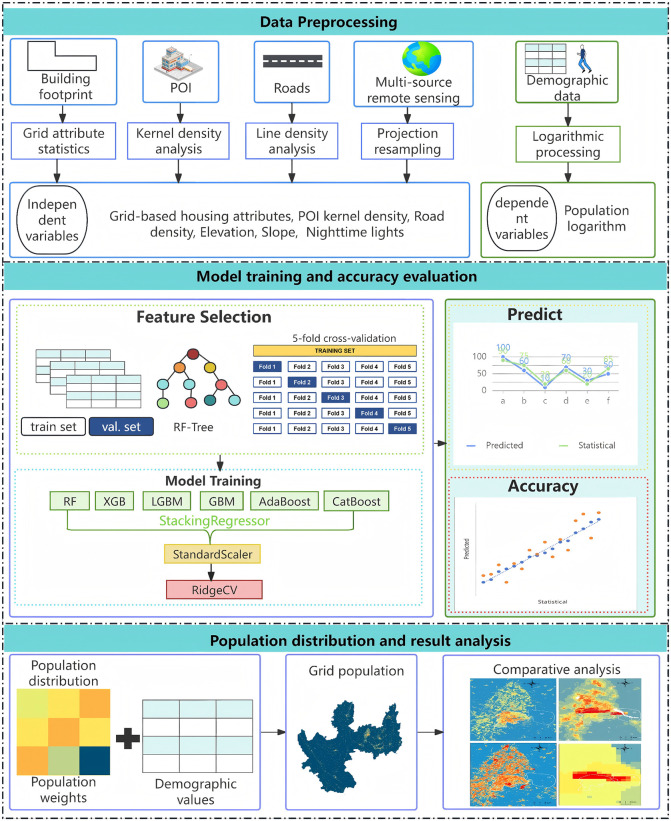
Overall Process of the Methodology.

The overall methodological process is shown in Fig 1,which includes three main parts: Data Preprocessing, Model Training and Accuracy Evaluation, and Population Distribution and Result Analysis(Fig 1).

- Data Preprocessing: All data are standardized to the WGS84 coordinate system. Attribute statistics are conducted for grid cells and administrative units, including the count of various POIs, building areas, heights, floor counts, building ages, slope, elevation, nighttime light intensity, and road network density. For administrative units, statistics are calculated at the administrative village scale, with the average values for relevant attributes. Based on the population data of each statistical unit and POI information, highly correlated POI types, building attributes, and multi-source remote sensing features are selected to construct population attraction indicators.
- Model Training and Accuracy Evaluation: Based on the Stacking ensemble strategy, multiple learners are integrated, with training and estimation carried out at the administrative unit and grid levels. The model’s training accuracy is evaluated.
- Population Distribution and Result Analysis: First, population weights are constrained by assigning a weight of 0 to grids without buildings. The predicted grid results are then used as population weights. Using Partition Density Mapping, the census data is redistributed based on the weights, resulting in a population spatial distribution dataset for Yuxi City at a 50m resolution. The final results are analyzed.

### 3.1. Population distribution indicator construction

#### 3.1.1. Selection of high-correlation POI types.

Selecting POI types that have a high correlation with population distribution is an essential task in population spatialization [23]. Feature selection is a crucial preprocessing step in machine learning, aiming to identify the most critical features for model prediction from the original feature set. This enhances the model’s performance and interpretability, reduces algorithm runtime, and increases learning efficiency [24] Feng [25]. Random Forest (RF) is an ensemble learning method that constructs multiple decision trees for training and prediction [26]. Its core principle is to combine the Bagging algorithm with CART decision trees, creating multiple sub-datasets through random sampling with replacement,training independent decision trees, and aggregating results via voting or averaging.Random Forest can handle high-dimensional data, offering high predictive accuracy and stability while maintaining low computational complexity. It is robust to outliers and noise, and can assess feature importance, providing valuable insights for feature selection [27].

Using administrative villages in Yuxi City as the statistical unit, with population data as the target variable and the count of various POIs as the feature values, a Random Forest algorithm is used to train the model and extract feature importance. The importance of each feature is calculated based on its frequency of use in decision tree split nodes and its explanatory power for the target variable. The top M features are selected, and Pearson correlation coefficients are calculated to eliminate highly correlated features. Finally, the remaining features are chosen to ensure high importance and minimal multicollinearity, which could negatively impact the model. Exhaustive searches for all feature combinations are performed using 5-fold cross-validation to select the best-performing feature combination, ensuring the POI types highly correlate with population distribution.

#### 3.1.2. Population distribution feature extraction.

This study applies kernel density estimation (KDE) to convert the POI population attraction indicators into continuous surfaces and extracts feature values for different POI types. The best search radius for each feature is obtained through correlation analysis. Kernel Density Estimation (KDE) is a method that uses a kernel function to interpolate spatial discrete points, converting them into continuous surfaces, as expressed by Equation (1):


$$\text{f}(\text{x})=\sum_{\text{i}\;=\;\text{1}}^{\text{n}}\frac{\text{k}}{{\text{r}}^{\text{2}}}(\frac{\text{x}\;-\;{\text{x}}_{\text{i}}}{\text{r}})$$ (1)

where *f(x)* is the estimated feature value of POI at point *x*; *r* is the search radius; *x*_*i*_ represents the spatial location of POIs; *n* is the number of POIs within the search radius; and *k* denotes the kernel function.

The method presented in this paper only considers the density feature extraction method based on the spatial distribution and quantity differences of POIs (Points of Interest) and does not account for the heterogeneity of population service and attraction capabilities of different POI facilities, such as the differences in population attraction between large integrated supermarkets and community convenience stores. In addressing the homogeneity of POI modeling in existing population regression models, one approach matches Weibo check-in data with POIs to construct a population attraction index, providing insights for addressing POI heterogeneity. However, these check-in data are often from different sources than the matched POIs (e.g., POIs from Weibo check-ins may not align with those from Gaode), leading to a mismatch between POIs and check-in data, which introduces bias into the population attraction index. Additionally, check-in POIs are mostly related to attractions, dining, shopping, and other entertainment types, making it difficult to accurately depict the attraction of other types of POIs on the population. Another method involves matching mobile location data with POIs to build a population attraction index. However, mobile location data based on phone usage involves personal privacy concerns and is difficult to obtain, limiting its broad application potential. From the perspective of individual POI categories, both large integrated supermarkets and community convenience stores are classified as shopping services, with a count of 1 for each, and their population attraction weights are considered equivalent. This contradicts the common belief that the population attraction of large integrated supermarkets is far higher than that of community convenience stores. However, comparative studies show that large integrated supermarkets are usually located in the commercial centers of cities, with many small shops surrounding them, and the POI kernel density in the area of the supermarket location is much higher than that of community convenience stores. Thus, using POI kernel density methods, we can see that different scales of POIs within the same type exhibit differences in population attraction. Similar patterns are observed for other types of POIs. Therefore, this paper adopts a simplified, unweighted POI kernel density analysis method for evaluation.

Although this study adopts a simplified, unweighted approach, we argue that incorporating a weighted KDE in future work would better capture the heterogeneity of POIs. By assigning weights based on POI type or scale, model accuracy could be substantially improved, particularly in high-density areas such as commercial centers. Moving forward, we plan to integrate additional dynamic data (e.g., mobile positioning data and real-time traffic) and further refine POI analysis using the weighted KDE to enhance adaptability across different regions and data types.

### 3.2. Population distribution weight modeling

Since true population data at the grid level is difficult to obtain directly, population spatialization methods typically involve constructing modeling factors at the administrative unit level and the weight relationship between these factors and population. This relationship is then transferred to the grid level, where it serves as the population weight when redistributing census data. This methodology allows for the estimation of population for each grid cell.

The modeling process is based on administrative village-level data, where average building area, height, number of floors, age, high-correlation POI density, elevation, nighttime light intensity, and road density are selected as independent variables, and the log of population for each administrative village is used as the dependent variable. The independent variable data is standardized to minimize the influence of different scales. Stacking ensemble strategy is employed, integrating multiple base learners, including Random Forest, XGBoost, LGBM, GradientBoosting, AdaBoost, and CatBoost models. In the model training and evaluation phase, machine learning models are trained to capture the nonlinear relationship between independent and dependent variables. K-fold cross-validation is used for accuracy validation and model performance evaluation.

**Fig 2:**
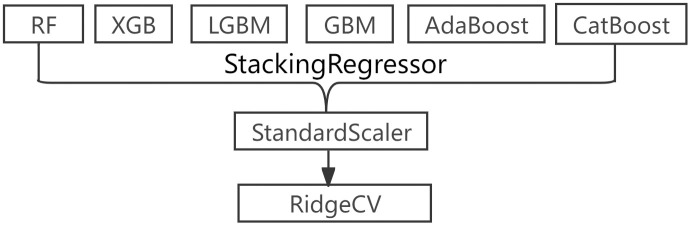
Ensemble Learning Model Architecture.

Due to the inherent limitations in the design principles of various machine learning algorithms, a single model often fails to fully capture the multidimensional patterns in complex data, leading to insufficient generalization performance due to excessive bias or variance [28]. Ensemble learning effectively reduces the risk of misselection from a single model by strategically combining the strengths of multiple models, thus minimizing the possibility of falling into local optima and improving prediction stability and accuracy. Stacking, as a hierarchical ensemble strategy, focuses on extracting multi-view features from the data using base learners, followed by optimal fusion of these features through a meta-learner, ultimately enhancing the model’s overall information processing capability. The Stacking ensemble learning architecture built in this paper is shown in Fig 2, and its design logic and performance optimization mechanisms are as follows:

Base Learner Selection Logic: This paper selects six models as base learners: Random Forest, XGBoost, LGBM, GradientBoosting, AdaBoost, and CatBoost. The core consideration is to balance model diversity with information complementarity. From an algorithmic perspective, Random Forest implements double random sampling of both samples and features based on the Bagging strategy and is adept at handling non-linear interactions in high-dimensional data. XGBoost and LGBM optimize the decision tree splitting process through the gradient boosting framework, using regularization to control complexity and histogram algorithms to improve efficiency. GradientBoosting and AdaBoost enhance the learning of misclassified samples through different loss function weight update strategies. CatBoost introduces a category feature automatic encoding mechanism to reduce preprocessing dependency. This combination retains the tree model’s ability to capture non-linear relationships (ensuring overlapping behavior), while also constructing diversity through differences in sampling strategies, optimization targets, and feature processing methods. As a result, the base learner group can cover various data patterns. When some models exhibit bias on specific samples, the complementary predictions from other models can reduce overall errors.

Meta-Learner Selection of RidgeCV: This paper uses Ridge Regression with cross-validation (RidgeCV) as the meta-learner, considering both theoretical suitability and empirical effectiveness. From a theoretical perspective, when the prediction results of the base learners are used as meta-features, multicollinearity can arise due to the correlations between the models (for example, different Boosting models may generate similar responses to key features). RidgeCV’s L2 regularization mechanism can reduce the impact of collinearity by shrinking the coefficients, ensuring parameter stability. Additionally, the regularization term effectively prevents overfitting, which is especially useful in scenarios with high-dimensional meta-features (with six base learners corresponding to six input dimensions). From an empirical perspective, the cross-validation (CV) mechanism allows the model to adaptively select the optimal regularization parameter λ, dynamically balancing bias and variance. RidgeCV has demonstrated better generalization ability than standard linear regression and unregularized Ridge regression on various datasets.

Model Diversity and Performance Synergy: The performance gain of Stacking fundamentally stems from the organic combination of base learner diversity and synergy. Diversity manifests in dimensions such as model structure (Bagging vs Boosting), feature sensitivity (e.g., Random Forest is more robust to noise, XGBoost is more sensitive to key features), and error distribution (different models exhibit error regions in the sample space). These differences allow the meta-learner to focus on more reliable predictive signals through weight allocation. Synergy, on the other hand, is reflected in the fact that the collective predictions of the base learner group cover feature combinations that a single model cannot reach. The meta-learner aggregates scattered useful information through regularized linear fusion, ultimately achieving “1 + 1>2” performance enhancement. When some base learners exhibit bias due to local optima, the complementary predictions from other models can be reinforced by the meta-learner’s coefficient adjustment, thereby reducing overall prediction error.

Secondary Training Set Construction: To avoid overfitting signals from the base learners being passed to the meta-learner during training, this paper adopts a 5-fold cross-validation strategy to generate meta-features. The initial training set T is randomly divided into five subsets, T_1_-T_5_. For each base learner, four subsets (T-T_i_) are used for training, and the remaining subset T_i_ is used for validation to obtain the predicted results Y_i_ for T_i_. After traversing all subsets, the six base learners generate complete prediction matrices, which, together with the original target variable, form the secondary training set T_S_. The meta-learner RidgeCV is then trained on TS using regularized linear regression, integrating the predictions from the six base learners, and ultimately constructing a high-performance Stacking ensemble prediction model.

### 3.3. Population distribution

Population distribution involves allocating the census population of administrative villages to various grids. Density zoning mapping is a commonly used method in the spatialization of population data, where census data is redistributed to grids based on weights [8]. In this study, 50m grid data for each independent variable is used as input data for the ensemble learning model. After processing through the ensemble learning model, the predicted population logarithm for each grid is obtained as the dependent variable. The total predicted population logarithms of all grids within a given administrative village are calculated. The proportion of the population logarithm for a specific grid relative to the total population logarithm of all grids within the administrative village represents the population proportion weight W_j_ of that grid within the administrative village. The total population at the administrative village level is then allocated to each grid, as shown in Equation (2):


$${\text{D}}_{\text{ij}}=\frac{{\text{P}}_{\text{i}}\times {\text{W}}_{\text{j}}}{\sum_{\text{j}=\;\text{1}}^{\text{k}}{\text{W}}_{\text{j}}}$$ (2)

Where:

*i* represents the administrative district number;

*j* represents the pixel number within the administrative district;

*k* represents the total number of non-empty pixels within the administrative district;

*W*_*j*_ represents the weight of the pixel;

*P*_*i*_ represents the total census population within the iii-th administrative district;

*D*_*ij*_ represents the population within a single pixel.

### 3.4. Accuracy evaluation method

The study uses census data at the administrative village level as the training set and employs k-fold cross-validation to evaluate the accuracy of different models, with k = 10 In k-fold cross-validation, the original training set is divided into k subsets, with k − 1 subsets used as the training set and 1 subset used as the validation set. This process is repeated for all subsets, and the average of the k evaluation errors is taken as the final evaluation error. The error evaluation metrics include Mean Absolute Error (MAE), Mean Squared Error (MSE), Root Mean Squared Error (RMSE), and the Coefficient of Determination (R^2^). The calculation formulas are as follows in equations (3)–(6):


$$\begin{array}{c} MAE=\frac{\text{1}}{N}\sum_{i=\text{1}}^{N}|X_{i}-Y_{i}| \end{array}$$ (3)

$$\begin{array}{c} MSE=\frac{\text{1}}{N}{\sum_{i=\text{1}}^{N}\left(X_{i}-Y_{i}\right)}^{\text{2}} \end{array}$$ (4)

$$\begin{array}{c} RMSE=\sqrt{\frac{\text{1}}{N}{\sum_{i=\text{1}}^{N}\left(X_{i}-Y_{i}\right)}^{\text{2}}} \end{array}$$ (5)

$$R^{\text{2}}=\frac{{\sum_{i=\text{1}}^{N}\left(X_{i}-\bar{Y}\right)}^{\text{2}}}{{\sum_{i=\text{1}}^{N}\left(Y_{i}-\bar{Y}\right)}^{\text{2}}}$$ (6)

Where:

*X*_*i*_ represents the iii-th predicted value in the training set;

*Y*_*i*_ represents the corresponding iii-th validation value in the validation set;

*N* represents the total number of validation values;

$\overset{―}{Y}$ represents the average of the validation values in the validation set.

## 4. Experiments and results

### 4.1. Results of highly correlated POI selection

**Fig 3:**
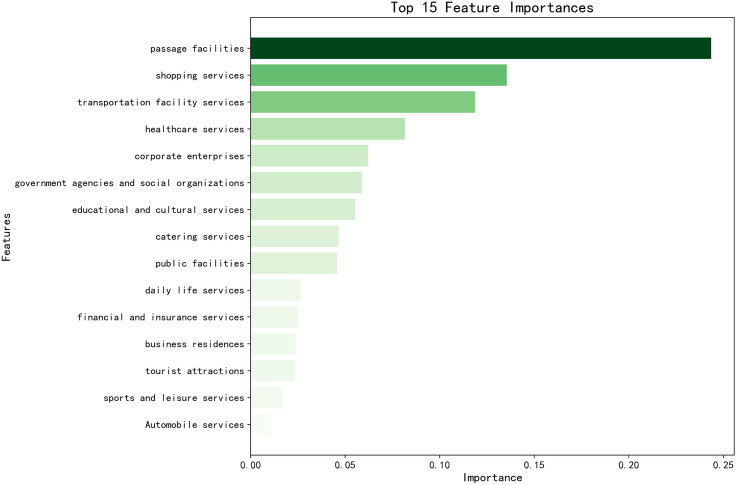
Ranking of the top 15 POI types based on their importance to population attraction.

A random forest algorithm was applied to train a dataset of 18 types of Points of Interest (POI) across 657 administrative villages in Yuxi City. Based on the frequency of each POI type’s use in decision tree split nodes and its explanatory power for the population size of the administrative villages, the importance of each POI type was calculated and ranked in descending order. The top 15 POI types are shown in Fig 3.

**Fig 4:**
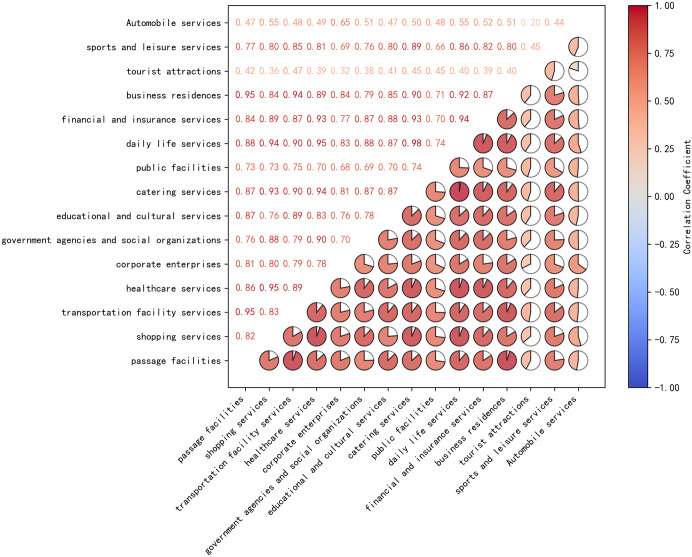
Pearson correlation coefficient matrix between the top 15 POI types based on importance.

**Fig 5:**
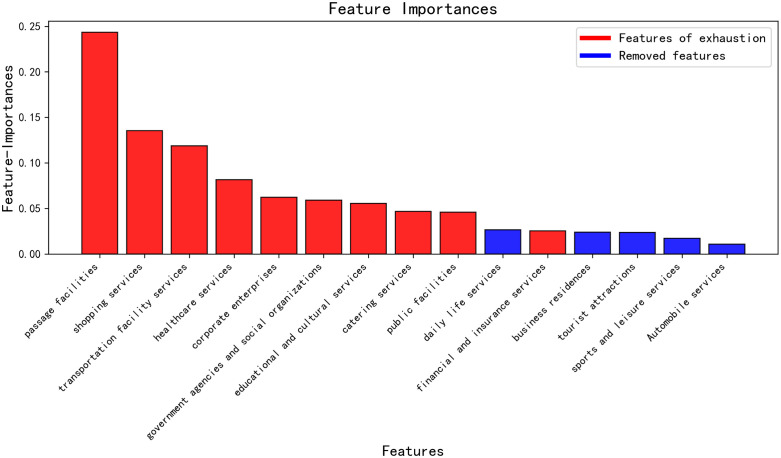
POI types with lower correlation (shown in red).

To address the issue of linear correlation between the top 15 POI types, the Pearson correlation coefficient matrix was calculated for the selected POI types, as shown in Fig 4. The filled circles represent the absolute values of the correlation coefficients, with colors distinguishing positive (red) and negative (blue) correlations. Redundant features with high correlation (i.e., |r| > 0.95) were removed to reduce the feature set, preserving the most informative features. From the top 15 POI types, those with lower correlation were retained, ultimately selecting 10 POI types. This process ensured that the selected features had high importance and minimized the effects of multicollinearity. A bar chart was created to display the importance of the features, with colors indicating the retained POIs (red) and the removed ones (blue), as shown in Fig 5.

For the selected 10 POI types, ArcGIS 10.2 software was used for kernel density estimation for each POI type. Then, the importance coefficient of the kernel density to the population log was calculated using a random forest model. The weight for each POI type was calculated according to Equation (7). Finally, based on the weights of each POI type, the 10 POI kernel density layers were merged into one, and the average POI kernel density for each administrative village was calculated. To account for the effect of search radius, a proximity analysis was performed to determine the search radius range for the POIs. Kernel density analysis was then carried out with certain step sizes. Correlation coefficients between the extracted features and population log were calculated for different search radii, and the optimal search radius was determined based on the correlation. According to the study by Yang X et al. (2019), the correlation between POI kernel density and population log was highest at a 500m radius.


$${\text{W}}_{\text{i}}=\frac{\%\text{IncMS}{\text{E}}_{\text{i}}}{\sum_{\text{k}=\text{1}}^{\text{10}}\%\text{IncMS}{\text{E}}_{\text{k}}}$$ (7)

*W*_*i*_ represents the weight of the i-th POI kernel density; *%IncMSE*_*i*_ is the %IncMSE value of the i-th POI kernel density; the sum of the *%IncMSE* values for the 10 POI kernel densities is calculated. The *%IncMSE* value represents the percentage increase in mean squared error (MSE), which measures the importance of each variable in regression tasks using models like random forests. A higher *%IncMSE* value indicates greater importance.

### 4.2. Population distribution weight modeling results

The modeling process involved establishing a population distribution weight model based on administrative village data. The average values for building height, number of stories, building age, and floor area for both urban residential and non-residential buildings, as well as for rural residential and non-residential buildings, were calculated for each administrative village. Additionally, the average values for nighttime light intensity, road network kernel density, POI kernel density, elevation, and slope were used as independent variables in the model. The abbreviations for the independent variables of building attributes are shown in Table 2.

**Table 2:** The abbreviations for the independent variables of building attributes.

Abbreviation	Full name
NFURB	the number of floors in urban residential buildings
FAURB	the floor area of urban residential buildings
HURB	the height of urban residential buildings
AURB	the age of urban residential buildings
NFUNRB	the number of floors in urban non-residential buildings
FAUNRB	the floor area of urban non-residential buildings,
HUNRB	the height of urban non-residential buildings
AUNRB	the age of urban non-residential buildings
NFRRB	the number of floors in rural residential buildings
FARRB	the floor area of rural residential buildings
ARRB	the age of rural residential buildings
ARNRB	the age of rural non-residential buildings
FARNRB	the floor area of rural non-residential buildings
NFRNRB	the number of floors in rural non-residential buildings

**Fig 6:**
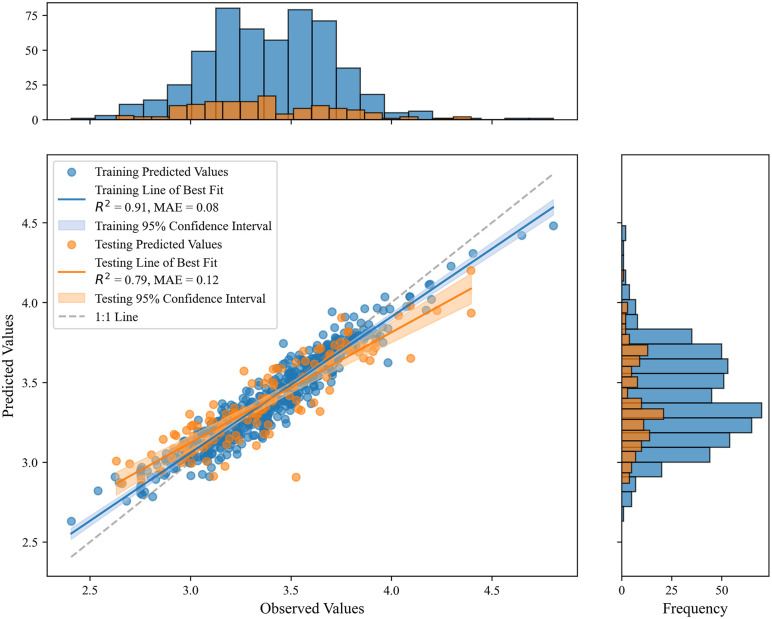
Results of the ensemble learning mode.

The population logarithm of each administrative village was taken as the dependent variable. Random forest, XGBoost, LGBM, Gradient Boosting, AdaBoost, and CatBoost models, along with an ensemble model, were applied to build the relationship model between independent and dependent variables. The results of the ensemble learning model are shown in Fig 6.

### 4.3. Accuracy validation

The accuracy validation results of various machine learning models on both the training and test sets are shown in Table 3. On the test set, the Stacking ensemble model demonstrated the best performance, achieving an R^2^ of 0.7969, which is approximately 1.3% higher than the second-best Gradient Boosting model (0.7867). Additionally, Stacking had the lowest MSE (0.0267), RMSE (0.1633), and MAE (0.1238) among all models, with superior error control across all metrics. Compared to the worst-performing AdaBoost (R^2^ = 0.7181), Stacking outperformed by 10.97% in R^2^, with all three error metrics showing a reduction of over 15%, indicating a significant advantage.Gradient Boosting performed best on the training set (R^2^ = 0.9323), but its performance on the test set declined noticeably, showing signs of overfitting. XGBoost (test set R^2^ = 0.7855) and LightGBM (R^2^ = 0.786) performed similarly, both outperforming Random Forest (R^2^ = 0.7688) and CatBoost (R^2^ = 0.7591). CatBoost’s relatively weaker performance is likely due to the limited presence of categorical features in the data, which prevented its symmetric tree structure from being fully utilized.Random Forest demonstrated relatively robust generalization ability, with its MAE increasing by 41.2% on the test set compared to the training set, a smaller increase than AdaBoost (82.6%). However, the RMSE increased significantly by 42.9%, indicating sensitivity to extreme values. AdaBoost performed the worst on both the training and test sets, confirming the model’s lack of robustness when dealing with noisy data.Stacking, by fusing the outputs of multiple heterogeneous base learners and performing weighted integration through a meta-model, achieved the best overall performance on the test set. Its R^2^ decay rate between the training and test sets (approximately 11.6%) was lower than most single models, demonstrating better generalization ability and stability. The simultaneous reduction in error metrics and more balanced error distribution further highlights the significant value of the Stacking ensemble strategy in complex predictive tasks.

**Table 3:** Comparison of accuracy results of various machine learning models on the training and test sets.

Model	R²		MSE		RMSE		MAE	
	Training Set	Test Set	Training Set	Test Set	Training Set	Test Set	Training Set	Test Set
Random Forest	0.8505	0.7429	0.0154	0.0322	0.1241	0.1794	0.0987	0.1419
XGBoost	0.9297	0.7871	0.0072	0.0266	0.0851	0.1632	0.0677	0.1227
LightGBM	0.883	0.8035	0.0121	0.0246	0.1098	0.1568	0.0834	0.119
Gradient Boosting	0.9376	0.7794	0.0064	0.0276	0.0802	0.1662	0.0648	0.1264
AdaBoost	0.7544	0.7131	0.0253	0.0359	0.1591	0.1895	0.129	0.1508
CatBoost	0.8585	0.764	0.0146	0.0295	0.1207	0.1718	0.0965	0.1345
Stacking	0.9122	0.7911	0.0091	0.0261	0.0951	0.1617	0.0757	0.1243

### 4.4. Feature interpretability

SHAP values are a game-theoretic approach used to explain the prediction results of individual samples. They measure the contribution of each feature to the prediction result for that sample. In the plotted SHAP feature chart, the color represents the magnitude of the feature value, with blue indicating lower values and red indicating higher values. The points in the chart are shaped like a bee swarm, which helps visualize the distribution of data points and avoids overlap. Each point represents the SHAP value of a data sample, and the distribution of points shows the impact of that feature on the model output. The horizontal axis represents the contribution of SHAP values, which indicates the degree to which each feature influences the model’s prediction. Positive values indicate that the feature increases the predicted value, while negative values indicate that the feature decreases the predicted value. The vertical axis lists the features used in the model. Some charts include bar plots that represent the average absolute SHAP value for each feature, indicating its importance. The longer the bar, the greater the impact that feature has on the model’s prediction.

**Fig 7:**
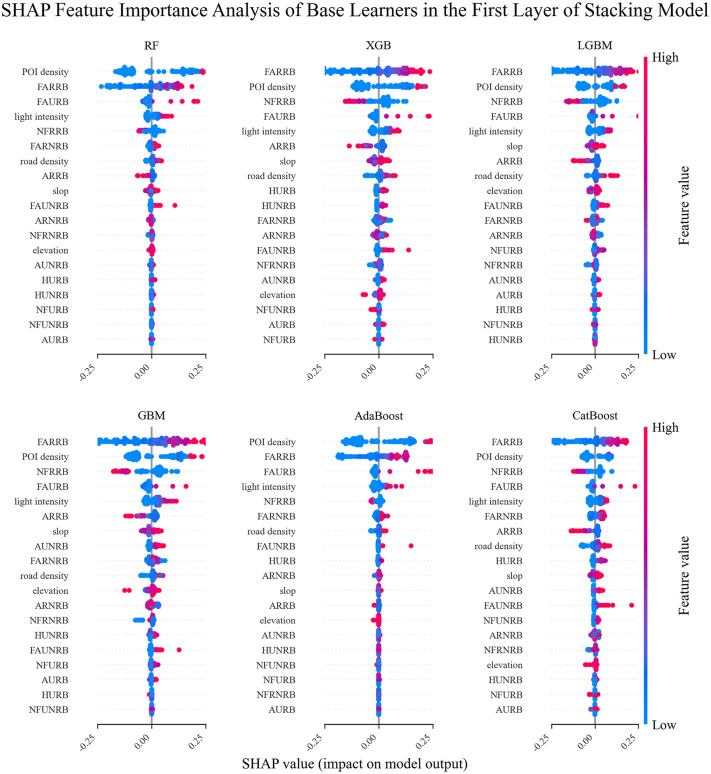
SHAP feature importance plots for 6 base learners.

The SHAP feature importance bee swarm plot for all base learners in the Stacking model is shown in Fig 7. It can be observed that the SHAP explanations for each base learner are different, which is due to each base learner working independently and having different preferences for the features.

**Fig 8:**
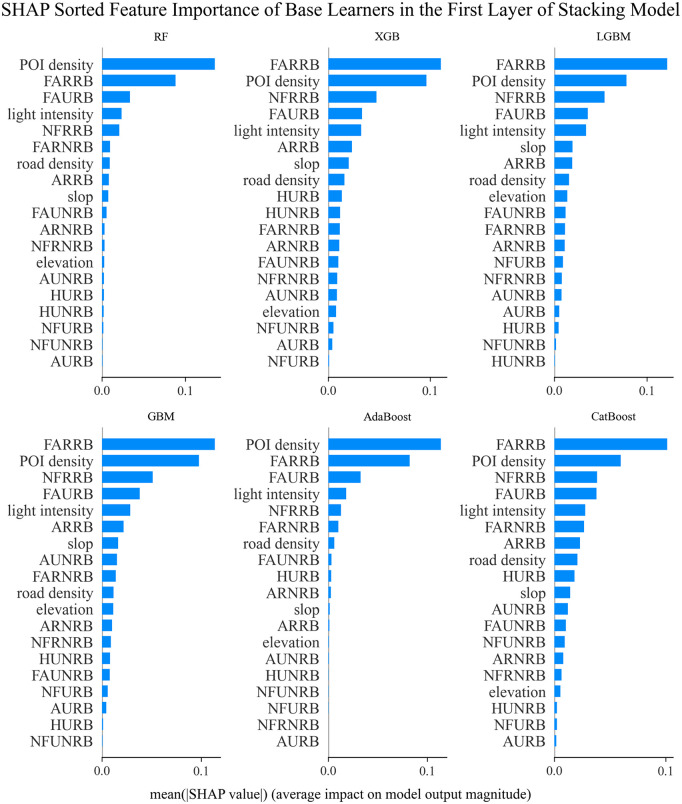
SHAP feature contribution ranking plots for 6 base learners.

SHAP feature contribution ranking plots for all base learners in the Stacking model are shown in Fig 8. These plots demonstrate the average influence of each feature’s importance for each base learner.

In this study, a stacked model was constructed to perform a multi-algorithm comparison analysis for the population distribution weight model of administrative villages. The SHAP feature importance distribution was explored for the first-layer base learners (including RF, XGBoost, LightGBM, AdaBoost, GBM, and CatBoost).In terms of commonality, the floor area of rural residential buildings (FARRB) and POI kernel density (POI density) are key features, ranking in the top 2 across all six base learning models, with SHAP values ranging from 0.15 to 0.25. This demonstrates that the floor area of rural residential buildings has universal explanatory power for population distribution, while POI kernel density shows the highest positive impact in RF, XGB, and AdaBoost, validating the positive correlation between the distribution of various facilities and population aggregation. At the same time, the age of urban residential buildings (AURB) shows a negative impact in all models, indicating a consistent negative correlation between older buildings and population size.In terms of model specificity, tree-based models like RF and XGB focus more on geographical features such as building height and elevation, while gradient boosting models like LGBM and CatBoost are more sensitive to spatial features such as POI kernel density and rural building attributes. AdaBoost and GBM demonstrate unique capabilities in analyzing building age.This heterogeneity in feature attention patterns validates the synergistic enhancement mechanism of the stacked model. By integrating the complementary strengths of different learners in spatial distribution analysis, geographic feature extraction, and temporal pattern capture, the stacked model effectively enhances the representation capability for complex spatial prediction tasks (such as administrative village population distribution weight prediction), providing a richer perspective for understanding the relationship between population distribution and building-related features.

**Fig 9:**
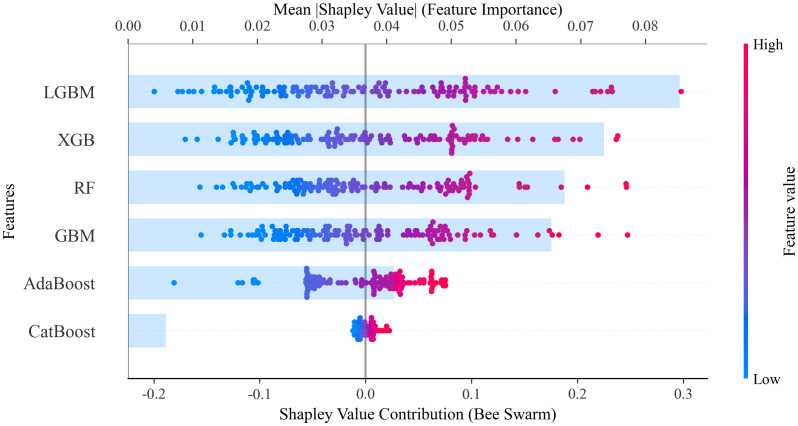
SHAP summary plot and feature contribution ranking plot of the meta-learner.

As shown in Fig 9, visualization based on the hexbin plot reveals significant heterogeneity in the impact of each base learner’s prediction on the meta-learner’s decision. The absolute range of Shapley value contributions is [−0.2, 0.3], indicating that the predictive outputs from different base learners influence the ensemble decision in a directional manner (either positively or negatively). This asymmetry in feature distribution suggests that the meta-learner’s decision mechanism is not simply an additive combination of predictions but involves a nonlinear relationship. The ranking of average feature importance (Mean Shapley Value) in descending order is: LGBM(0.086)> XGBoost(0.073)> RF(0.068)> GBM(0.064)> AdaBoost(0.042)> CatBoost(0.006).This quantification indicates that gradient boosting-based LightGBM and XGBoost dominate the ensemble system, with their weighted coefficients for predictions significantly higher than those of the other learners.

**Fig 10:**
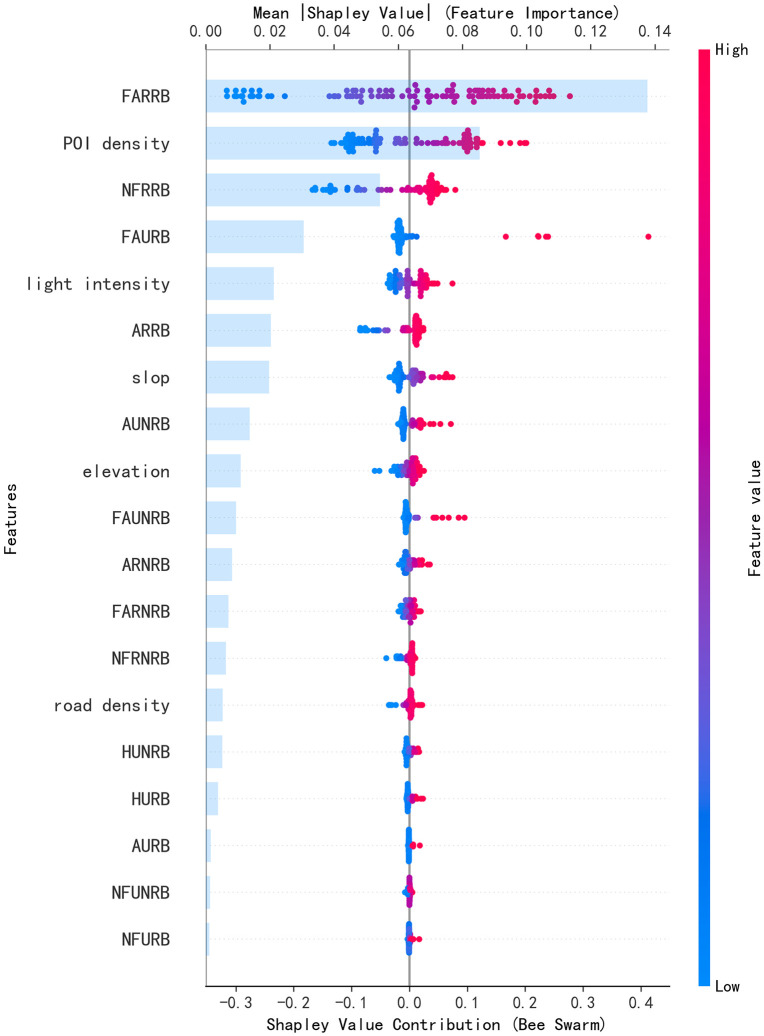
SHAP summary plot and feature contribution ranking plot for the overall contribution of the Stacking model.

In Fig 10, Shapley values quantify the nonlinear impact mechanisms of multidimensional features on the population count of administrative villages. The results provide valuable insights into the understanding of urban and rural population distribution patterns. Observing the range of feature contributions reveals significant asymmetry, with positive driving effects notably stronger than negative suppression effects.

Among the core positive driving factors, the floor area of rural residential buildings exhibits the strongest explanatory power. The red distribution trend indicates that as the value of this indicator increases, its marginal contribution to population growth becomes more significant, confirming the foundational role of the expansion of rural residential land in promoting rural population aggregation. POI kernel density, representing the concentration of urban functions, shows an increasing contribution as the characteristic value rises (with dense red areas), demonstrating that the spatial concentration of commercial and service facilities has a multiplier effect on population attraction. Notably, the contribution intensity of rural residential building height exceeds that of similar urban indicators, revealing the special significance of vertical expansion in rural living spaces for population carrying capacity, which is linked to the spatial intensive use under rural land policy constraints. Secondary influencing factors exhibit significant regional differentiation. Nighttime light intensity, as a proxy for economic activity, shows a pronounced positive contribution, indicating that the increase in brightness above a certain threshold has a nonlinear accelerating effect on population attraction. The positive contribution of rural residential building age suggests the stabilizing effect of rural housing renovation on population control. Among the terrain features, slope exhibits a dual effect, possibly reflecting the promoting role of moderate topographical undulations on population settlement, while excessive undulation may constrain it. The four major plateau lakes in Yuxi City (Fuxian Lake, Xingyun Lake, Qilu Lake, and Yangzonghai) have small slopes in the lake areas, which are unsuitable for human habitation, while areas with steeper slopes are less favorable for human survival, leading to a lower population. Urban non-residential building indicators generally show weak correlations, with the Shapley values for features such as building age and height concentrated in the ± 0.05 range, indicating that urban non-residential functional spaces have limited direct impact on community population distribution. It is noteworthy that the contribution of road density spans both positive and negative values, which may reveal an optimal density threshold for road network development, beyond which excessive construction leads to a population dispersion effect.

The construction of the ensemble model effectively integrates the feature interpretation advantages of each base learner. Through SHAP value-weighted analysis, it was found that the standard deviation of the combined importance of rural residential building area, POI kernel density, and urban residential building height decreased from 0.05 in the individual models to 0.02, significantly improving the stability of feature explanation. This multi-model comparative analysis method provides cross-validation at the algorithmic level for feature selection in the ensemble model, enhancing the scientific rigor of research on the mechanisms influencing population spatial distribution.

**Fig 11:**
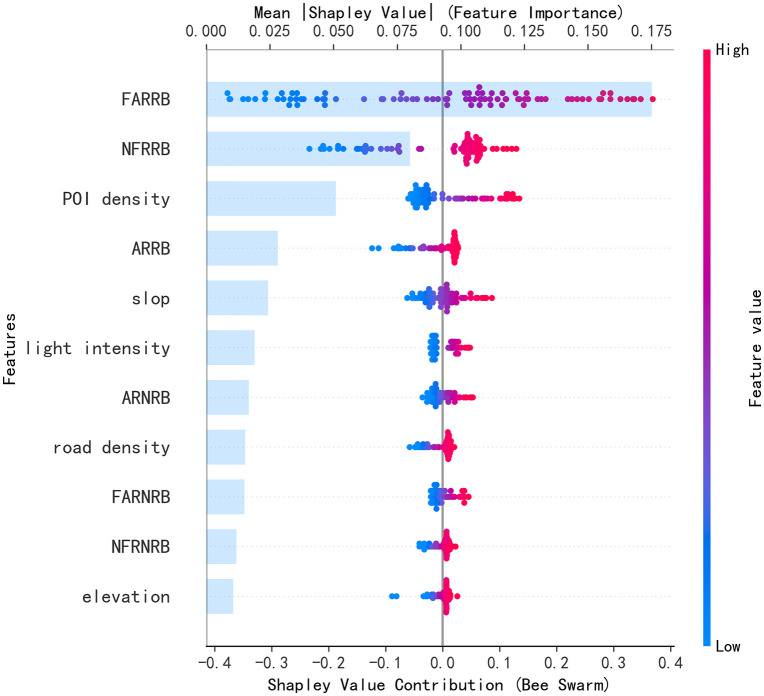
SHAP Summary Plot and Feature Contribution Ranking Plot for the Overall Contribution of the Stacking Model in Rural Areas.

**Fig 12:**
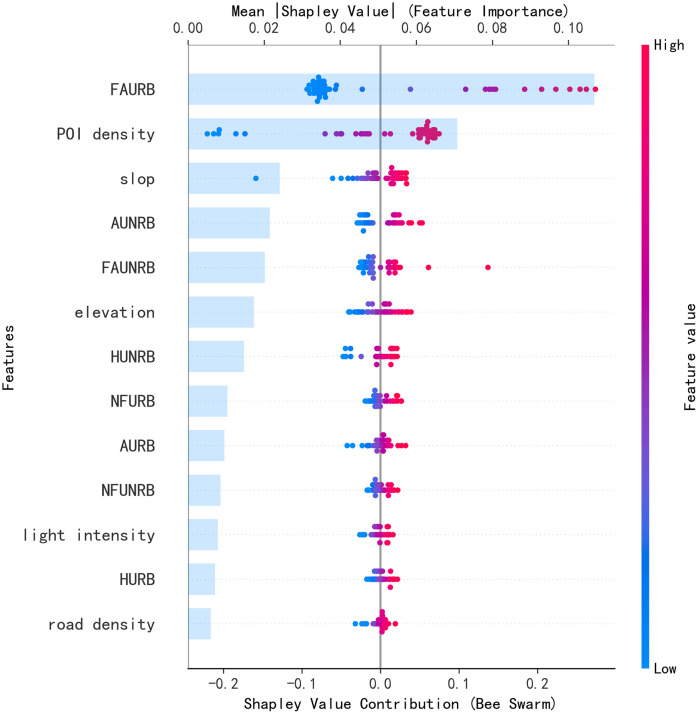
SHAP Summary Plot and Feature Contribution Ranking Plot for the Overall Contribution of the Stacking Model in Urban Areas.

To compare how the SHAP results vary between subregions (urban vs. rural) and thereby reveal the spatial heterogeneity of variable effects, we categorized the data for urban and rural areas and separately computed the magnitudes of feature contributions and their impacts on the predictions. The results are shown in Figs 11 and 12.In rural areas, the floor area of rural residential buildings (FARRB) and the number of floors (NFRRB) are the core factors affecting the population of administrative villages. Larger floor areas and more stories directly reflect rural housing capacity, which may attract population aggregation or reflect existing high–population-density demand. Points-of-interest (POI) density also plays a role. Building age (ARRB, ARNRB), slope (slop), elevation, and features related to rural non-residential buildings likewise have significant impacts on population, most of them negative: when buildings are older, slopes steeper, elevations higher, or size-related indicators for non-residential buildings are unfavorable, populations tend to be smaller.

In urban areas, FAURB (floor area of urban residential buildings) and POI density are the key factors influencing population—the former by providing sufficient living space and the latter by reflecting abundant commercial and service facilities—both exerting significant positive effects. By contrast, slop (slope) has a clear negative effect due to topographic constraints. Factors such as AUNRB (construction year of urban non-residential buildings), FAUNRB (floor area of urban non-residential buildings), and HUNRB (height of urban non-residential buildings) also affect population through channels such as industrial employment and functional carrying capacity. These results reveal the spatial heterogeneity of the determinants of population distribution across urban and rural areas.

The differences in SHAP results between urban and rural areas essentially reveal the fundamental disparities in the population distribution logic under the binary urban-rural development pattern. This deep significance can be explored from three dimensions: functional positioning, mobility logic, and governance orientation.

From the perspective of functional positioning, rural population distribution is still highly dependent on the physical carrying capacity of residential space. The core influence of rural residential building area (FARRB) and the number of floors (NFRRB) reflects that rural population aggregation is still fundamentally based on “residential space supply” logic. In the context of relatively singular rural industrial functions, residential capacity directly determines the upper limit of population carrying capacity. Negative factors such as building age and slope highlight the rigid demand for “livable basic conditions” in rural areas. In contrast, urban areas exhibit a shift toward a “demand-driven” population distribution model, where the dual key roles of POI density and urban residential building area (FAURB) reflect that sufficient living space is a basic guarantee, while the abundance of commercial, service, and other POI facilities represents core attractions such as employment opportunities and living convenience. This aligns with the urban role as an “economic and service hub.”Moreover, the significant impact of non-residential building characteristics (AUNRB, FAUNRB, etc.) in urban areas further emphasizes the deep connection between urban population distribution and industrial layout. The spatial distribution of industrial employment opportunities has become a key variable in guiding urban population concentration.

This urban-rural distinction also implies the underlying logic of population mobility: rural populations are more influenced by “residential and survival conditions,” while urban populations are driven by “development opportunities and quality of life.” This finding provides precise guidance for population control strategies: rural areas should prioritize improving living conditions and creating a more livable environment, while urban areas should optimize POI distribution and balance residential and non-residential functions to achieve coordinated population and functional distribution.

### 4.5. Population spatialization simulation results

**Fig 13:**
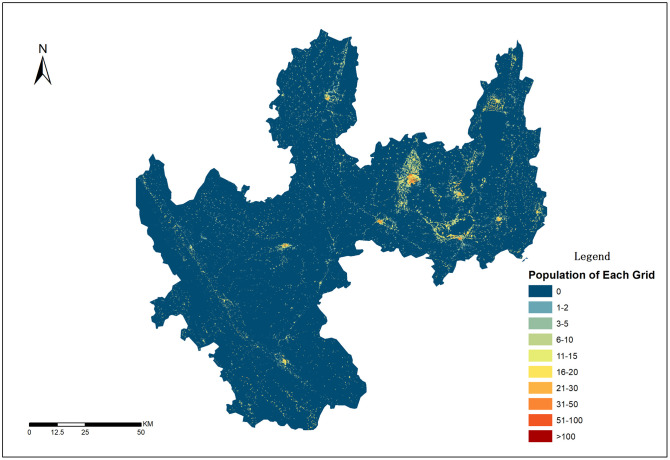
Spatial Distribution and Description of Yuxi City’s 2020 Population at 50m Resolution.

Using the predicted population-distribution weight layers, dasymetric mapping was performed, and the authors produced a 50 m-resolution spatial distribution map of the 2020 administrative-village census population in Yuxi City, as shown in Fig 13.

By analyzing the population spatialization distribution results derived from ensemble learning modeling methods, it was found that the population peaks are concentrated in the city center of Yuxi, with a peak value of approximately 800 people per grid. The higher the urban level, the larger the radius of the population peak area. For instance, the population peak circle in Hongta District (population density > 50 people/grid) has a radius of about 3 km, while Jiangchuan District’s peak circle has a radius of about 1 km, Tonghai County’s peak circle spans approximately 3 km, and Chengjiang City’s peak circle covers around 1 km. Hongta District, as the political, economic, and cultural center of Yuxi, has a population significantly higher than that of other districts and counties. This region, benefiting from its advantageous transportation location (only 88 kilometers away from Kunming, the provincial capital of Yunnan) and a developed industrial system (which contributes 40% to Yuxi’s GDP), creates a strong attraction, presenting a monopolar agglomeration pattern.

The “Three Lakes” region (Fuxian Lake, Xingyun Lake, and Qilu Lake) in Jiangchuan District, Chengjiang City, and Tonghai County, based on its developed agricultural base, tourism resources, and transportation advantages, has formed a ring-shaped population agglomeration zone around the lakes, which, although covering less than 10% of the city’s area, houses over 40% of the population. This region exhibits distinct “core-periphery” distribution characteristics and strip agglomeration patterns. From a spatial perspective, population distribution is significantly influenced by terrain conditions, economic development levels, and transportation networks, forming a three-dimensional distribution model with the basin area as the core and the mountainous areas as the periphery, extending along transportation routes and lake basins.

In contrast, areas like Yuanjiang County and Xinping County in the western mountainous gorges, which occupy 42.3% of the total area, have a population density of less than 60 people per square kilometer. This “dense east, sparse west” distribution pattern closely aligns with the distribution of arable land resources and the historical timeline of development. In summary, Yuxi City’s population distribution demonstrates a “single-core dominance, strip extension, and basin agglomeration” composite feature, with spatial differentiation resulting from the combined influence of natural geographical constraints, economic gradient differences, and policy guidance.

### 4.6. Results comparison and analysis

**Fig 14:**
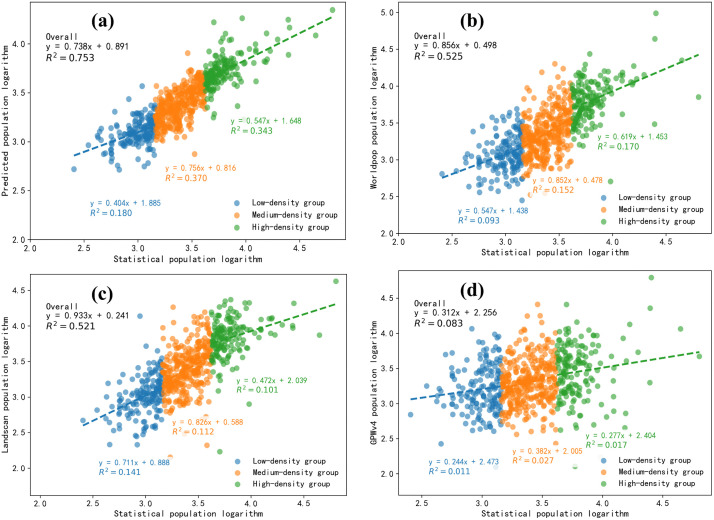
Validation of Population Prediction Results at the Administrative Village Level for Four Datasets, (a) A linear fit plot of the log of village statistical population versus the simulation results of the validation set of the spatialized model of ensemble learning, (b) A linear fit plot of the log of village statistical population versus the log of the population from the WorldPop dataset, (c) A linear fit plot of the log of village statistical population versus the log of the population from the landscan dataset, (d) A linear fit plot of the log of village statistical population versus the log of the population from the GPWv4 dataset.

To refine the comparison of data accuracy, the training samples were randomly divided into three folds, with two folds used as the training set and one fold as the prediction set, iterating until all samples were used. The simulation results of the validation set of the spatial model based on ensemble learning, the logarithms of statistical populations from the WorldPop, LandScan, and GPWv4 datasets, were linearly fitted with the logarithms of the seventh national census population of communities/administrative villages. Fig 14 shows the logarithmic linear relationship between the statistical population of 2020 and the predicted population, WorldPop population, LandScan population, and GPWv4 population. Based on the population density calculated from the seventh national census data, the 657 communities/administrative villages in Yuxi City were divided into three groups: a low population density group (blue), a high population density group (green), each accounting for 25% of the total sample, and a medium population density group (orange), accounting for 60% of the total. The closeness between the predicted and statistical values is represented by the fitted line, where a slope closer to 1 and a coefficient of determination closer to 1 indicate more accurate predictions. The overall prediction result in this study is 0.753, which is the highest accuracy among the four datasets. The WorldPop and LandScan datasets have overall accuracies of 0.525 and 0.521, respectively, with both showing similar accuracy. The GPWv4 dataset has the lowest overall accuracy of 0.083, much lower than the other three datasets. The main reason is that the GPWv4 population dataset uses a 1000-meter grid, while the predicted population dataset in this study uses a 50-meter grid, the WorldPop dataset uses a 100-meter grid, and the LandScan dataset uses a 90-meter grid. The area of a single grid in the GPWv4 dataset is 100–400 times larger than the other three datasets. Large-scale grid population datasets inherently experience averaging effects within individual grids when distributing population, which cannot capture the heterogeneity of the population’s spatial distribution. Additionally, the areas of administrative villages in Yuxi City range from 0.5 to 100 square kilometers, with 10 administrative villages having areas smaller than 1 square kilometer, much smaller than the area of a single grid in the GPWv4 dataset. There are also cases where one grid belongs to multiple administrative villages. These factors lead to significant discrepancies when using large-scale population datasets to estimate the population within a specific administrative region. The prediction results in this study show an accuracy improvement of 43.43% over the WorldPop dataset and 44.53% over the LandScan dataset, while a comparison with the GPWv4 dataset is not meaningful due to the difference in grid size. The prediction results, along with the WorldPop and LandScan datasets, all underestimate population in high-density areas and overestimate population in low-density areas.

**Fig 15:**
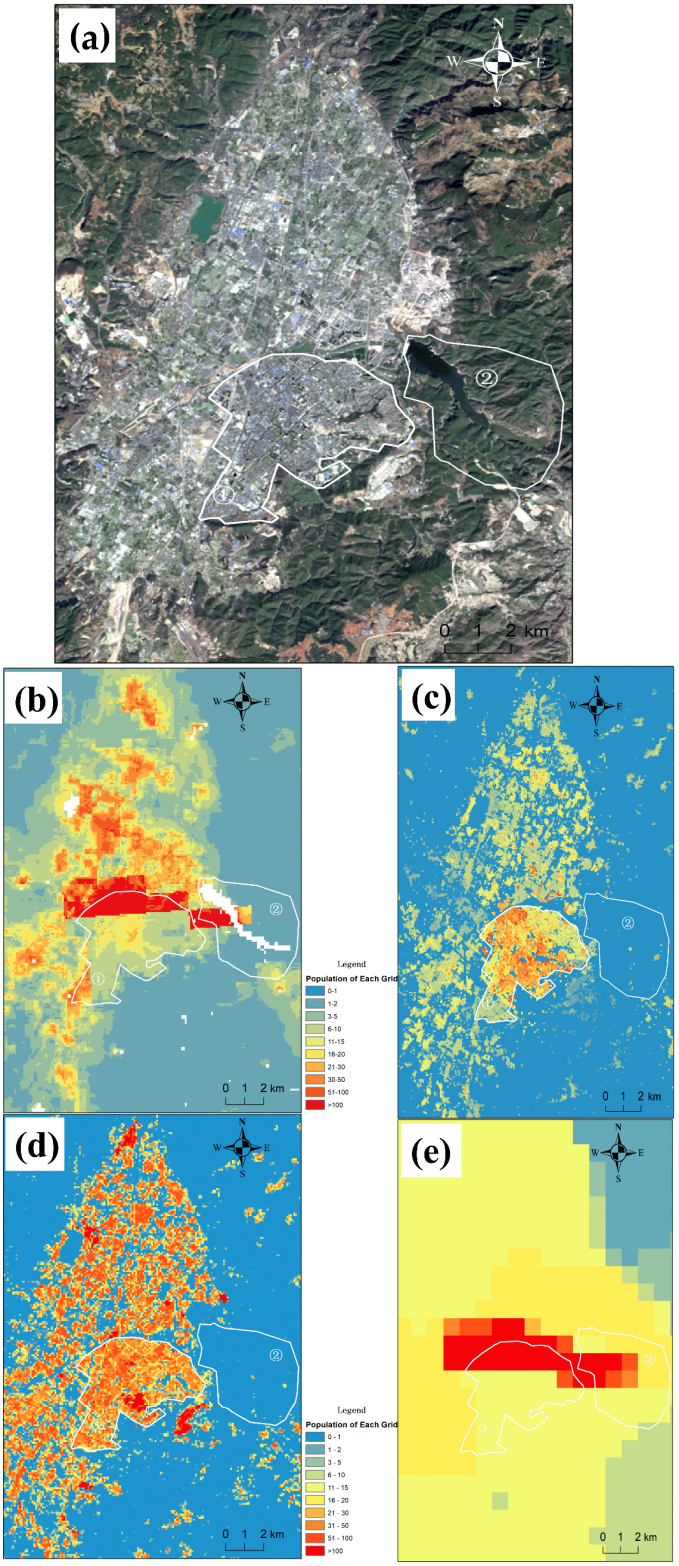
Population spatial distribution maps for the central urban area of Yuxi derived from different data sources.

To examine differences in spatial visualization between the results of this study and mainstream gridded population datasets, two areas with contrasting population densities in Hongta District, Yuxi City, were selected. Landsat imagery and spatial distribution maps derived from the WorldPop, LandScan, and GPWv4 population datasets were compared with the Stacking ensemble-learning simulation results proposed in this paper (Fig 15). Subfigure (a) shows the Landsat imagery of the urban area of Yuxi City; subfigure (b) presents the spatial distribution from the WorldPop dataset; subfigure (c) displays the spatial distribution from the ensemble-learning population dataset; subfigure (d) shows the spatial distribution from the LandScan dataset; and subfigure (e) illustrates the spatial distribution from the GPWv4 dataset.

In Figs (a), (b), (c), (d), and (e), the area outlined by the white lines, labeled as region ①, is the central area of Yuxi City. From Fig (a) (Landsat imagery), it is evident that the area has dense buildings, developed commercial zones, and numerous high-rise buildings. In Fig (b), the WorldPop population dataset and in Fig (e), the GPWv4 dataset show high-density population grids concentrated in the northern and northern peripheral regions of area ①, which clearly do not match the high-density urban building clusters shown in the Landsat imagery. In Fig (c), the ensemble learning population simulation results appear more reasonable, with high-density population grids primarily concentrated within the boundaries of area ①, displaying a scattered distribution that aligns well with the high-density building distribution in the urban area, with noticeable terrain features at the edges. In Fig (d), the LandScan population dataset shows high-density population grids distributed more uniformly across area ①, failing to capture the spatial variability within the area. The Landsat imagery reveals that the urban high-density building clusters in region ① are concentrated in the central part, while the buildings in the northeast and southwest are relatively sparse compared to the central area.

In Figs (a), (b), (c), (d), and (e), the area outlined by the white lines, labeled as region ②, is the location of the Dongfeng Reservoir in the suburban area of Yuxi City. From Fig (a) (Landsat imagery), it is clear that region ② mainly consists of the reservoir’s water storage area and surrounding green spaces, with a few scattered buildings. In Fig (b), the WorldPop population dataset and in Fig (e), the GPWv4 dataset show a large concentration of high-density population grids in the western part of region ②, and the reservoir area also displays many medium-density population grids, which clearly does not correspond with the building distribution shown in the Landsat imagery. In Fig (c), the ensemble learning population simulation results and in Fig (d), the LandScan population dataset show more reasonable outcomes, with no high-density population grids in region ②, only a few medium-density grids scattered around, and no population in the reservoir area, aligning with the building distribution in the imagery.

In conclusion, the ensemble learning population simulation results in Fig (c) perform the best in region ① compared to the other three datasets. In region ②, the results are as excellent as the LandScan population dataset and better than the WorldPop and GPWv4 datasets.

## 5. Discussion

Machine learning models have become a common method for processing large and complex datasets in the era of big data, effectively overcoming the limitations of traditional regression models, which struggle to capture nonlinear relationships. As population distribution is influenced by various factors, such as natural geography, socioeconomics, housing, and transportation networks, the relationships between input variables are complex. Consequently, research has gradually adopted different machine learning models for spatial population prediction. For example, YE Tingting et al [28], Tan Min et al [15], and Liu Yi et al [17] used random forest models for population spatialization simulations at three scales: national, urban agglomeration, and city. These studies achieved high prediction accuracy, but significant differences were observed in simulating high, medium, and low population density units, possibly overlooking spatial heterogeneity in population distribution in certain areas. Similarly, due to the lack of real and accurate population grid data, the simulation accuracy of various machine learning models in population spatialization is difficult to quantify. The risk of instability is higher for single models, whereas ensemble learning models, with their generalization properties, expand the hypothesis space and balance the diversity of different machine learning models.

In this study, a Stacking ensemble learning strategy was applied to the population spatialization simulation for Yuxi City, using Random Forest, XGBoost, LGBM, GradientBoosting, AdaBoost, and CatBoost models as primary learners, and Ridge regression (RidgeCV) as the secondary learner. Ridge regression efficiently handles multicollinearity in the independent variables, stabilizes coefficient estimation, and prevents overfitting through regularization, thus improving the generalization ability and prediction accuracy of the model. This method effectively mitigates the overestimation of high population values observed in single models. Compared to the WorldPop dataset, the ensemble learning model in this study significantly improves population peak values in urban centers, displaying a scattered distribution and considering the distribution of buildings, avoiding the issue of large population distributions in areas without buildings.

In today’s era, the increasing human demands and the rising threat of seismic disasters to human life and safety have gained unprecedented attention. Population spatialization distribution affects the earthquake disaster risk levels, and the population spatialization simulation process must continuously adapt to changing distribution patterns. Early studies typically used nighttime lights, vegetation indices, and elevation data for population spatialization simulations. However, these remote sensing data cannot directly represent the spatiotemporal characteristics of human activities. With the rapid development of internet and communication technologies, urban activities now generate virtual reflections, and the use of location data from social media and geographic spatial big data has become widespread, providing new solutions for high-resolution population data spatialization. For example, Ye Tingting et al. [28] introduced POI (Points of Interest) data into population spatialization simulations, finding that it significantly improved the WorldPop dataset’s underestimation of urban populations and overestimation of rural populations.

The building data and POI social perception big data used in this study have high variable importance, ranking among the top input variables. However, these data have a relatively slow update frequency and are semi-static. Dynamic location data, which directly records human activity locations, contains rich spatiotemporal semantic information and provides a potential solution to the limitations of static data in explicitly representing population size and activity intensity. For example, mobile positioning data and Tencent location big data generated from social media visits. Given the key role of dynamic social perception big data in this research, future studies will delve deeper into its temporal and semantic attributes, refining population spatialization grids from both temporal and spatial dimensions, and providing more accurate foundational data for disaster risk assessment, urban planning, and emergency response.

Population distribution is also influenced by surrounding facilities. POI data contains rich spatial semantic information and can be used for urban functional zoning and identification, reflecting human activity patterns to some extent. According to the first law of geography, the closer two things are, the more strongly they are related. Therefore, grid units closer to POIs are considered to have a higher impact on population distribution than those farther away [29]. In this study, we used building patch data alone for weight constraints in grid population weighting, without considering the impact of distance between buildings and different types of POIs on population distribution. Future research could extract distance features by calculating the Euclidean distance between various building types within grid cells and the nearest POIs, exploring the population allocation weights for each grid. Moreover, Euclidean distance does not account for obstacles affecting path accessibility, which could lead to ineffective weight distribution in some areas. Large obstacles (such as rivers and reservoirs) and connecting infrastructure (e.g., bridges) can alter local spatial connectivity and impact population distribution. A non-Euclidean filtering method that considers connectivity should be developed to correct spatial autocorrelation of population weights.

### 5.1. The applicability of the method in this paper

The “Multi-source Data Fusion + Stacking Ensemble Learning + Building Weight Correction” methodology proposed in this study has a significant universal framework characteristic. Its core logic is to improve population spatialization accuracy through the integration of multi-source data (social perception big data, housing building data, remote sensing data, etc.) and the construction of ensemble learning models. This basic framework can be adapted to different urban forms and regional characteristics. In terms of framework universality, the method has modular advantages: the variable selection logic can be flexibly replaced according to regional features, the base learner combination can be dynamically adjusted based on data types (e.g., adding temporal models in high-dynamic regions), and the regularization mechanism of the secondary learner ensures stability under different data quality conditions. This framework can be applied to large cities with significant multi-center characteristics, underdeveloped regions with scarce data, and medium-sized prefecture-level cities, with only targeted adjustments needed based on specific scenarios. Additionally, the core influencing factors of population distribution (natural geography, socioeconomics, buildings, transportation, etc.) have common patterns in different regions, providing a foundation for cross-regional application of the method. For example, the high building density and rich POI characteristics of population concentration areas in large cities, and the infrastructure distribution characteristics of core settlements in remote areas, can all be characterized through the data fusion logic and model structure within the framework.

The method in this study is highly adaptable to data and can handle different data endowment scenarios through a stratified strategy. For data-rich mega-cities, dynamic data (e.g., mobile signaling, traffic flow) fusion can be enhanced, adding spatiotemporal sequence models and attention mechanisms to capture population spatiotemporal fluctuations. For medium-data prefecture-level cities, “Principal Component Analysis + Domain Knowledge Mapping” can be used to fill missing POI data, combined with remote sensing imagery to correct the precision of building data. For data-scarce county or rural areas, lightweight model variants can be constructed, simplifying the variable set (remote sensing baseline data + key infrastructure POIs), reducing the number of learners, and adjusting the loss function weights to decrease dependency on scarce data. Additionally, alternative data strategies (e.g., using land use type area to replace detailed building data) can further lower the application threshold. The transferability of this method relies on modular design and flexible adjustment mechanisms. In cross-regional applications, variable selection logic can be replaced based on target area characteristics (e.g., adding flow-related variables like metro stations in high-density cities), the base learner combination can be adjusted according to data types (e.g., introducing temporal models in dynamic regions), and stability can be ensured through the regularization mechanism of secondary learners (e.g., using ridge regression to handle multicollinearity). This “core framework fixed + module dynamic adaptation” approach provides a scalable technical path for regions with different data conditions. To address the overfitting risk in different regions, this study proposes targeted prevention strategies: for data-rich mega-cities, spatially stratified cross-validation (by functional zoning of the validation set) is used to avoid bias caused by homogenization of regional features; for regions with medium data volumes, POI attraction weight coefficients are recalibrated to reduce parameter fitting bias; and for data-scarce regions, regularization parameters are increased to reduce model complexity, while retaining robust learners (e.g., random forests) to ensure model stability with limited data.

Although applying this framework in different regions and under various data modalities has its advantages, it also faces numerous challenges. In megacities, despite the abundance of data, dynamic data (such as mobile phone signaling and traffic flow) come from multiple sources and are in heterogeneous formats. During data fusion, issues of inconsistent spatiotemporal benchmarks are likely to arise. Moreover, spatiotemporal sequence models and attention mechanisms have extremely high demands for computing resources, which may result in low training efficiency of the models. Additionally, when using spatially stratified cross-validation to divide the validation sets according to functional zones, if the boundaries of functional zones are ambiguous, it is prone to causing validation biases.When applying the method of “principal component analysis + domain knowledge mapping” to supplement missing POI data in prefecture-level cities, the subjectivity of domain knowledge may affect the accuracy of data completion. Furthermore, the effectiveness of using remote sensing images to correct the inadequate accuracy of building data is restricted by the image resolution and the complexity of ground features.When constructing lightweight model variants in county or rural areas, simplifying the variable set may lead to the loss of key information, and there is a lack of uniform standards for adjusting the weights of the loss function. In alternative data strategies, the correlation between land use type areas and detailed building data varies across different regions, which can easily affect the accuracy of the models.During cross-regional applications, significant differences in natural and socioeconomic conditions exist among different regions. The adjustment of variable screening logic and primary learner combinations relies on a profound understanding of the characteristics of the target regions, necessitating extensive experimentation and experience. The accuracy of the framework’s evaluation results will face challenges.

### 5.2. Analysis of systematic bias across models

The emergence of systematic bias across models is closely related to the interaction among data characteristics, model structures, and spatial attributes, with error estimation under sparse density being particularly critical. From the data perspective, the sparsity of POI data is one of the core drivers. In low-population-density areas, such as counties or rural regions, POIs are inherently scattered and tend to concentrate in a few core settlements, while key POI types such as retail services and transport facilities are underrepresented. Although the POI kernel density method can capture differences in attractiveness among POIs of the same type but different scales, it is prone to producing “pseudo–low density” under sparse conditions. When the number of POI samples is too small, kernel smoothing fails to faithfully reflect the actual population-carrying capacity of a region, weakening the association between model input features and the true population distribution, and thereby introducing bias.

From the perspective of model structure, ensemble learning improves generalization by combining multiple models, but in sparse-density environments it can amplify the inherent weaknesses of individual learners and ultimately lead to systematic bias. For example, base learners such as Random Forest and XGBoost tend to suffer from “underfitting” in data-sparse regions because of insufficient feature information, making it difficult to capture local patterns of population distribution. The secondary learner, ridge regression, can handle multicollinearity but cannot compensate for the deterioration in feature quality caused by sparse data. In addition, the study notes that overfitting control strategies differ across regions; for instance, in data-scarce areas, the set of model variables is simplified. While this reduces model complexity, it may also omit key features (such as medical and educational POIs in remote areas), leading to persistently underestimated population density in sparse regions. Such bias is then likely to be solidified in the final results through the weight allocation mechanism of the ensemble learning framework.

At the level of spatial characteristics, the “absence of spatial heterogeneity” in sparse-density areas further exacerbates errors. According to the first law of geography, spatial proximity implies stronger association, but in POI-sparse regions, distances between grid cells and POIs are prone to “distance distortion” due to the uneven distribution of POIs. Some grid cells, lacking effective nearby POIs, are forced to rely on distant POIs to compute distances, so that distance-based features can no longer accurately represent the determinants of population distribution. Meanwhile, the lack of non-Euclidean filtering, as mentioned in the study, is particularly problematic in sparse regions: barriers such as rivers and mountains further fragment already sparse POI spatial linkages, preventing the model from correctly identifying the influence of connectivity differences on population distribution. As a result, error estimates tend to exhibit “contiguous patches of bias” in space, and such bias is difficult to fully correct within the existing ensemble learning framework, becoming an important source of systematic inter-model deviation.

The proposed building-weight correction partially alleviates this issue, but its reliance on Euclidean distance is still constrained by geometric space assumptions and fails to account for the mitigating effect of path-based accessibility on error propagation in sparse areas. Future weighted kernel density methods need to incorporate non-Euclidean distance measures (such as network distance that accounts for road topology), and combine heterogeneous decay functions (e.g., power-law decay for commercial facilities and logistic decay for public service facilities), in order to construct a density-adaptive error-correction framework that can effectively suppress the systematic bias induced by sparse densities.

### 5.3. Exploration of future research directions

With the widespread application of location-based social networks (LBSNs), user POI visit data in LBSNs not only reflects users’ interest preferences but also implicitly captures the distribution characteristics of population activities in geographical space [30]. Therefore, combining POI visit information with population density is expected to offer new perspectives and more accurate analytical methods for population spatialization research. The following are several specific future research directions:

**Population Density Estimation Based on POI Visit Frequency**

The frequency of visits to POIs can serve as an important indicator of population activity intensity in a given area. By analyzing the visit frequencies of different POIs, the latent population density in that area can be inferred. For example, commercial centers, restaurants, and entertainment venues with high visit frequencies are typically located in densely populated regions. Future research could construct a model that combines POI visit frequency with geographic coordinates, using machine learning algorithms (such as random forests or deep learning models) to predict population density across different geographical areas. This method not only leverages large-scale data from LBSNs but also dynamically reflects changes in population distribution over time.

**Spatiotemporal Analysis and Dynamic Population Spatialization**

Population density is not static; it dynamically changes over time and space. By analyzing user visit patterns to POIs across different time periods, spatiotemporal dynamics of population distribution can be captured. For example, certain areas may experience high population density in the mornings on weekdays, but be relatively sparse on weekends. Future research could incorporate spatiotemporal analysis techniques, such as spatiotemporal clustering or spatiotemporal regression models, to more accurately describe population distribution across time and locations. This approach could provide more precise decision support for urban planning, traffic management, and emergency response.

**Multisource Data Fusion**

In addition to POI visit data from LBSNs, other multisource data can be integrated to enhance the accuracy of population spatialization. For instance, LBSN data can be combined with mobile communication data, traffic flow data, satellite remote sensing data, and more. These data sources complement each other, providing a more comprehensive view of geographic spatial information. For example, mobile communication data offers broader user coverage, while satellite remote sensing data provides geographic and land use information. By integrating multisource data, more accurate population spatialization models can be developed, reducing potential biases from relying on a single data source.

**User Behavior Patterns and Population Characteristics Analysis**

User behavior patterns in LBSNs (such as visit frequency, visit time, stay duration, etc.) not only reflect personal preferences but may also be related to the socio-economic characteristics of the population. For example, high-income groups may visit high-end restaurants and entertainment venues more frequently, while low-income groups may be more likely to visit public transportation stations and supermarkets. Future research could further explore the relationship between user behavior patterns and population characteristics. By constructing user profiles and behavior models, the accuracy of population spatialization can be enhanced. This method could provide more detailed population distribution information for social science research and policy development.

Through the exploration of the aforementioned research directions, combining POI visit information from LBSNs with population density can provide new methods and tools for population spatialization research. This not only contributes to improving the accuracy of population distribution predictions but also offers more scientific foundations for urban planning, resource allocation, and policy formulation.

## 6. Conclusions

This paper presents a methodology for population spatialization simulation that integrates social perception big data, housing attributes, and multisource remote sensing data using Stacking ensemble learning. The method: (1) characterizes the differences in spatial distribution and quantity of various POI types representing population numbers, selects highly correlated POI types that influence population attraction, and models the quantitative relationship between POIs and population distribution; (2) employs Random Forest, XGBoost, LGBM, GradientBoosting, AdaBoost, and CatBoost models as primary learners, with Ridge regression (RidgeCV) as the secondary learner, to construct a high-performance Stacking ensemble learning model. In the population allocation process, building information is incorporated to correct population distribution weights, preventing excessive dispersion. A 50m grid-scale population spatialization experiment was conducted in Yuxi City, and accuracy verification results show that the proposed population estimation model outperforms the WorldPop dataset and LandScan dataset by more than 40% in terms of estimation accuracy at the administrative village scale, with higher simulation accuracy for different population density areas. Compared to 100m population spatialization data, the 50m results exhibit finer population spatial features as portrayed by satellite imagery, with richer population distribution information that more closely aligns with actual population distribution characteristics. Furthermore, by measuring the SHAP feature contribution and importance of variable factors, it was found that housing building area, POI, and nighttime light intensity are the most important indicators of population distribution in Yuxi City, indicating a higher correlation of social factors in population spatial redistribution.

However, this method has the following limitations that need improvement: The study only considers the density feature extraction method based on the spatial distribution quantity differences of POIs, without accounting for the heterogeneity of population service and attraction capabilities of different POI facilities. For instance, while community convenience stores and large supermarkets are both classified as shopping POIs, they are counted as “1” in quantity/density indicators, even though their population attraction indices differ by orders of magnitude. This homogenization of the relationship between POIs and population could be addressed in future research by introducing more detailed dynamic data (e.g., mobile positioning data) into the model for population distribution simulations.

## Supporting information

S1 Dataset Key factors influencing the spatial distribution of population. (XLSX)
